# Charcot revived

**DOI:** 10.1055/s-0045-1804489

**Published:** 2025-06-11

**Authors:** Hélio A. G. Teive

**Affiliations:** 1 Universidade Federal do Paraná, Curitiba PR, Brazil.

In 2025, between the last day of June and the first week of July, the 200 ^th^ anniversary of the birth of Jean-Martin Charcot (1825-1893) will be celebrated in Paris, France. 1 The event has the official support of the International Society for the History of the Neurosciences and will be attended by several professors of neurology from around the world. 1 Dr. Olivier Walusinski is the Local Committee President of the symposium, which is officially sponsored by Mr. Emmanuel Macron, President of the French Republic. 1 The importance of Professor Jean-Martin Charcot, considered the founder of modern neurology, is unquestionable and recognized worldwide. In his fruitful 31-year career as a doctor at the Salpêtrière Hospital, between 1862 and his death in 1893, Charcot held several important positions. He began, together with Vulpian, in the anatomy laboratory. Then, in 1872, Charcot became a professor of pathological anatomy. The chair for the study of diseases of the nervous system was created in 1882, with Charcot as the Professor. 2 3 Over this extended period, Charcot described numerous neurodegenerative diseases, including amyotrophic lateral sclerosis (known as Charcot's disease), multiple sclerosis, and Charcot-Marie-Tooth hereditary neuropathy, among many other seminal contributions. 2 3 4 5 6 7 The Hospital de la Salpêtrière became known internationally as the Mecca of world neurology, and countless doctors from throughout the world visited and trained in Professor Charcot's neurology department. 2 3 4 5 6 7 The influence is particularly noteworthy of Professor Charcot and his entire group of disciples, many of whom were internationally renowned such as Pierre Marie, Joseph Babinski, and Gilles de la Tourette, on the dissemination of neurological knowledge and the creation of neurology schools around the world, especially in South America. 8 9 Among the founders of Latin America neurology services trained in the Salpêtrière Hospital, Paris, France, the names of José Ramos Mejia (Argentina – considered the first neurology center in Latin America); Augusto Orego Luco (Chile – recognized as the “Charcot of America”); Antonio Austregesilo and Enjolras Vampré (both from Brazil, in the states of Rio de Janeiro and São Paulo, respectively); Américo Ricaldoni (Uruguay); and Oscar Trelles Montes (Peru). 8 In this year commemorating the 200 ^th^ anniversary of the birth of Professor Jean-Martin Charcot, he is celebrated worldwide as the pioneer of modern neurology, and his substantial contributions to developing the neurology specialty continue to be acknowledged throughout the world. 7 8 The influence of the neurological school he founded and disseminated globally through his numerous disciples remains prominent today. Therefore, it is worth remembering and considering as current a few famous statements by Charcot's disciples, Pierre Marie and Edouard Brissaud, who wrote his obituary in *Revue Neurologique* in September 1893 under the title “Nécrologie J.-M. Charcot”. 10 11 The following are some excerpts from this obituary:

*“Professor Charcot is dead. The day after such a sudden catastrophe, we are stunned and are unable to pay appropriate tribute to such a master,”*

*“But we must speak about the neurologist, or rather, the creator of neurology. Before him was darkness and chaos. With him, clarity and order,”*

*“But how can we simply list Charcot's works? That would be to describe the history of neurology in its entirety,”*

*“His chair was the glory of our faculty and the jewel in his crown,”*

*“Who among his pupils could forget him? Recognition is the sweetest feeling,* ***”*** 10
